# Quinolone-fused cyclic sulfonamide as a novel benign antifilarial agent

**DOI:** 10.1038/s41598-018-30610-7

**Published:** 2018-08-13

**Authors:** Suprabhat Mukherjee, Nikhilesh Joardar, Shovan Mondal, Andrea Schiefer, Achim Hoerauf, Kenneth Pfarr, Santi P. Sinha Babu

**Affiliations:** 1 Department of Zoology, Visva-Bharati University, Santiniketan, 731 235 India; 2 Department of Chemistry, Syamsundar College, Shyamsundar, 713 424 India; 3 Institute of Medical Microbiology, Immunology and Parasitology, University Hospital Bonn, Bonn, Germany; 4 German Center for Infection Research (DZIF), D-53127 Bonn, Germany

## Abstract

Search of potent antifilarial drugs has been a major thrust area in tropical medicine research over the decades. Herein, we report 4,7-dimethyl-3,4,7,8-tetrahydro-3λ^6^-[1,2]thiazino[4,3-*f*]quinoline-3,3,8-trione (*8l*) as a new class of antifilarial agent which is extremely potent, with lethality against all the developmental stages (oocyte, microfilaria and adult) of the filarial parasite *Setaria cervi*. Molecular investigation on its mode of action revealed that *8l* is a typical inducer of reactive oxygen species that triggers oxidative stress inside the filarid and further signals induction of apoptosis by activating both intrinsic and extrinsic pathways. Moreover, *8l* is also active against *Wolbachia*, the essential endosymbiont of several human infectious filarids. Selective toxicity against filarial parasites and non-toxic nature in rat model were found as unique traits of *8l* to be a future medicine. Taken *en masse*, this maiden report on a novel quinolone fused cyclic sulfonamide presents a promising therapeutic lead for lymphatic filariasis in future.

## Introduction

Lymphatic filariasis (LF), caused by the infection of filarial nematodes is the second leading cause of long-term disability to date^1^. The filarial parasites reside in the body cavity of the vertebrate hosts where they parasitize the lymphatics, resulting in the obstruction of the lymphatic vessels, incompetence, lymphostasis, lymphatic dysfunctions (hydrocele and lymphedema), interstitial fibrosis and immunological dysfunctions leading to elephantiasis^1^. The disease also promotes susceptibility to opportunistic infections during the progression of lymphoedema to elephantiasis. All these pathological consequences majorly affect the ability of an affected individual to earn an income^1^. The current epidemiological map includes 55 countries with an estimated population of 1.23 billion patients^1^. LF accounts for 2.8 million disability adjusted life years (DALYs), excluding any impact of social stigma usually experienced by the patients and their caregivers^1^. Presently, global control program of LF includes preventive chemotherapy through mass drug administration (MDA) using albendazole (400 mg) together with ivermectin (150–200 µg/kg) or with diethylcarbamazine citrate (DEC) (6 mg/kg)^1^. These drugs are effective in reducing the microfilarial load from the bloodstream but have limited effects on the adult stage parasites. In addition, these three drugs induce systemic and inflammatory adverse reactions in patients that result several pathological outcomes. Moreover, there are increasing reports on the emergence of resistance and/or sub-optimal responses with these drugs are considered as added complication^2^. Chemotherapy using anti-wolbachial antibiotics (doxycycline) is another treatment option that target filarial endosymbiont, *Wolbachia* that play important roles in the life cycle, fertility, survival and pathogenesis of the parasite^3–5^. Chemotherapeutic trials with doxycycline (200 mg/day) resulted in elimination of *W. bancrofti* microfilariae, reduced activity of the adult worms, alongside improved clinical manifestation^6,7^. However, widespread use of this drug for community-based control is inhibited by the logistics of a quite prolonged course of treatment (usually recommended for 4–6 weeks) and contraindications in children and pregnant women^8^. Therefore, development and/or screening of new classes of antifilarial drugs have been a prime focus amongst filarial researchers. Numerous natural, synthetic and semisynthetic compounds have been proposed for their efficacy as antifilarial drugs out of which most of the compounds failed in the early phases of clinical trials owing to numerous drawbacks like limited efficacy against the adult stage parasite, poor absorption and serum availability, and most importantly adverse side effects^2^. Therefore, there is still a strong need for a molecule that could be used to treat filarial parasite as well as *Wolbachia* without hampering the physiological homoeostasis of the host.

Sulfa drugs are the heterocyclic sulfonamides found in nature and are known for their numerous pharmacological and medicinal properties^9–12^. In addition to the sulfa drugs, bioactive quinolone subunits are considered as very attractive scaffolds in medicinal research owing to their broad-spectrum biological activities such as antimicrobial, antimalarial, antineoplastic, antidiuretic, antiarrhythmic, insecticidal and sedative properties^13–15^. In this context, heterocyclic compounds with a benzosultam core have a wide spectrum of bioactivities, such as antileukemic, anticancer, antitumor, enzyme inhibition property, etc.^16–19^. Therefore, it is likely that synthesis of quinolone fused sultam derivatives can provide us a useful direction in antifilarial drug development. This thought has prompted us to develop a hybrid molecule, i.e. coumarin and quinolone fused benzosultam, which may serve as a potential antifilarial drug. Based on this postulation, a series of twelve coumarin and quinolone fused benzosultams were synthesized^20^ and tested for their efficacy in inducing mortality in the filarial parasite *Setaria cervi* as reported previously^21^. Amongst the tested compounds, a novel quinolone-fused cyclic sulfonamide (4,7-dimethyl-3,4,7,8-tetrahydro-3λ^6^-[1,2]thiazino[4,3-*f*]quinoline-3,3,8-trione) was found to exhibit the highest efficacy with a low degree of toxicity on non-targeted cells and tissues^21^. Herein, we describe the molecular mechanism of action of the novel sulfa drug, 4,7-dimethyl-3,4,7,8-tetrahydro-3λ^6^-[1,2]thiazino[4,3-*f*]quinoline-3,3,8-trione (named *8l*) that has been found to be potent against the filarial nematode but is non-toxic to mammalian hosts.

## Results

### 8l possess potential antifilarial activity

**Figure 1:**
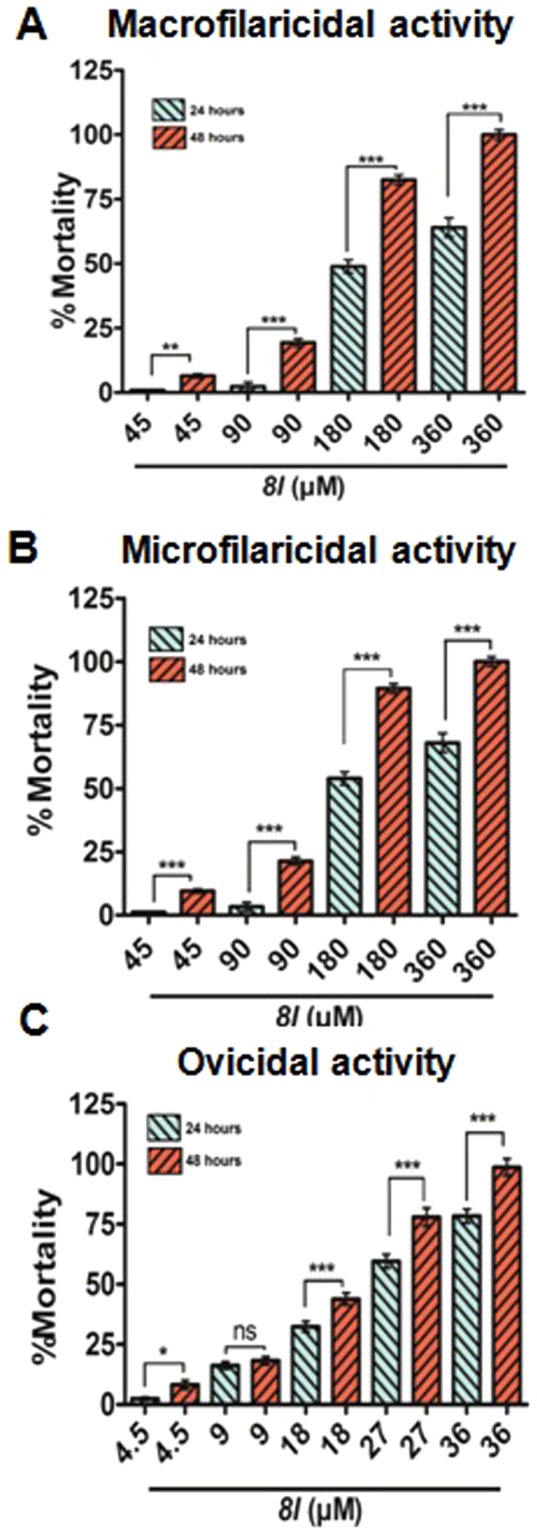
*In vitro* efficacy of *8l* against the developmental stages of *Setaria cervi*. Effect of *8l* on adult stage (**A**), microfilariae (**B**) and oocytes (**C**) of *S. cervi*. Mortality of all the developmental stages of the parasite *S. cervi* was determined by MTT assay and %mortality was calculated in comparison to the control. Each experiment was performed in triplicate and repeated for at least five times. Data were expressed as mean ± SD and *p < 0.05 was considered statistically significant (One Way ANOVA) as compared to control.

*8l* was found inducing lethality against all the developmental stages of *S. cervi* with the LD_50_ dose of 17.3 µM for oocytes, 166.9 µM for Mf and 281.4 µM for adult parasite (Fig. 1 and Supplementary Fig. 1). The activity of the compound followed a dose and time dependent trend. Intriguingly, activity of *8l* was higher than the standard filaricide ivermectin (Fig. 1 and Supplementary Fig. 1).

### 8l exerts antifilarial action through induction of apoptosis signaled from oxidative stress

**Figure 2:**
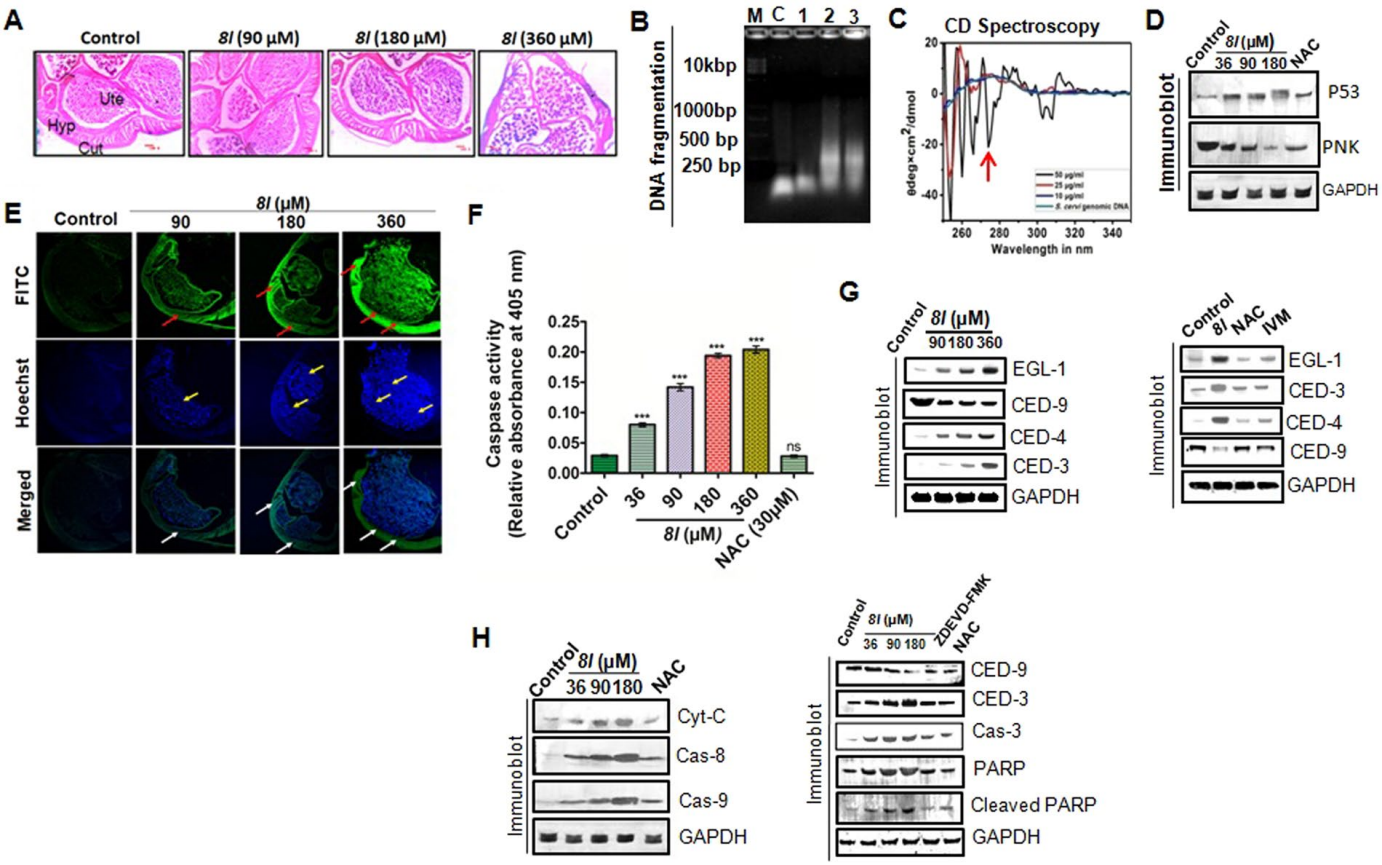
Molecular events behind the macrofilaricidal activity of *8l*. (**A**) Hematoxylin-eosin staining showing dose dependent changes in tissue architecture. (**B**) *8l* induces fragmentation in genomic DNA. (**C**) CD spectrum of genomic DNA of *S. cervi* in presence and absence of *8l*. The negative peak demonstrating *8l*-DNA interaction is indicated by the red arrow. (**D**) *8l* upregulates p53 protein but suppresses PNK expression. (**E**) Immunohistochemical staining showing expression of CED-3 in *S. cervi* tissue by green fluorescence of FITC (Upper panel). Hoechst stained micrographs showing chromatin condensation by blue staining (Middle panel). Merged images are given in the lower panel. (**F**) Caspase activity in *8l* treated parasites. Each data represents three independent experiments which were repeated for at least five times. ^*^p < 0.05 (One Way ANOVA) was considered statistically significant as compared to control. (**G**) Immunoblotting of apoptogenic protein showing *8l* induced apoptosis through activation of EGL-1-CED-9-CED-4-CED-3 pathway in adult *S. cervi* (left panel). Effects of ROS scavenger and standard filaricide Ivermectin on the activation of CED pathway. (**H**) Alteration in the expression of the signaling molecules of intrinsic and extrinsic pathways of apoptosis. Abbreviation: Cut, Cuticle; FITC, Fluorescein isothiocyanate; Hyp, Hypodermis; Ivm, Ivermectin; NAC, N-acetyl-cysteine; Ute, Uterus.

*8l* found to induce significant mortality in adult filarids (Fig. 1A) which prompted us to study the underlying mechanism behind the macrofilaricidal efficacy. Histological examination of control and *8l* treated parasites revealed damages in the epithelial lining and muscle layers (longitudinal and hypodermal), shrinkage and disintegration of cuticle alongside the syncytial hypodermis and disintegrated chromatin structure (Fig. 2A). Fragmentation of genomic DNA was also evident in *8l* treated adult *S. cervi* (Fig. 2B). Therefore, an obvious question arose about the possibility of physical interaction between *8l* and parasite DNA. This postulation was clarified by CD spectroscopy (Fig. 2C). A negative signal (between 250 nm to 320 nm) in the CD spectrum indicated direct physical interaction between *8l* and DNA especially at the minor groove (Fig. 2C).

In eukaryotic cells, any degree of damage to DNA increases the level of p53. Herein, a dose dependent upregulation of p53 protein was recorded in treated *S. cervi* (Fig. 2D). Under homeostatic condition, DNA damage recruits repair enzymes amongst which polynucleotide kinase (PNK) is a crucial player that recognizes damaged DNA and facilitates reconstruction of phosphodiester linkage^22^. However, we observed that *8l* treatment downregulated PNK expression and this might have caused suppression of DNA repair in treated parasites (Fig. 2D). These responses collectively indicated that *8l* could trigger apoptosis in *S. cervi*.

This assumption was verified by immunostaining of CED-3 (effector caspase of nematode) and Hoechst staining of histological sections of *S.cervi* (Fig. 2E). Dose dependent upregulation in CED-3 expression, mostly in the cuticle and hypodermis, was evident in *8l* treated parasite (Fig. 2E). The same was observed in the embryos in the uterine tube of treated *S. cervi* (Fig. 2E). We also detected an increasing trend in the number of condensed nuclei (a marker of apoptosis) in the treated parasites with the increase of treatment dose (Fig. 2E). Induction of apoptosis was further inferred from the increasing trend in caspase activity (Fig. 2F). Furthermore, a dose dependent up-regulation of the pro-apoptotic proteins EGL-1, CED-4 and downregulation of the anti-apoptotic protein CED-9 were found in *8l* treated parasites (Fig. 2G). These alterations primarily govern the induction of the active CED-3 that in turn facilitates the death of the worm (Fig. 2G). Appearance of cleaved PARP upon *8l* treatment (Fig. 2H) indicated the activation of intrinsic pathway of apoptosis in *S. cervi* alongside CED pathway^23^. In addition, upregulation in the expression of inducer and executioner proteins viz. cytochrome c and caspase-8, 9, 3 further evidenced the activation of intrinsic and extrinsic pathways (Fig. 2H; left panel). Diminished downstream response (PARP cleavage) in presence of caspase inhibitor (Z-DEVD-FMK) (Fig. 2H; right panel) indicated a putative crosstalk between CED-3 and caspase-3 pathways. Interestingly, administration of N-acetyl cysteine (NAC), a ROS scavenger, prevented the induction of apoptotic processes (Fig. 2H; right panel). Therefore, involvement of oxidative stress in *8l* induced apoptosis was studied.

**Figure 3:**
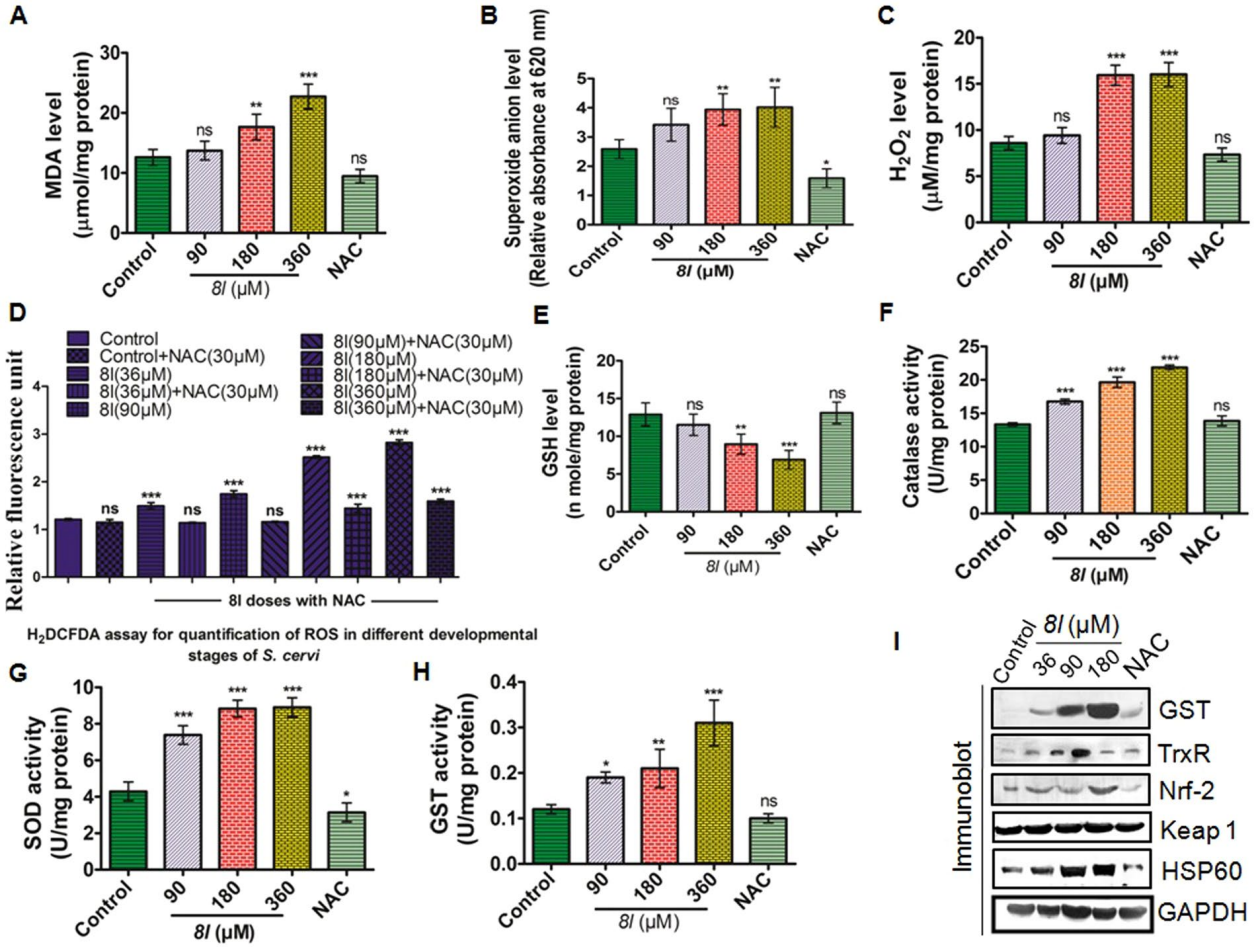
*8l* induces oxidative stress in *S. cervi* adults. *8l* treatment result increased (**A**) MDA level; (**B**) generation of Superoxide anion; (**C**) H_2_O_2_ production and (**D**) total ROS. *8l* results depletion of (**E**) reduced glutathione level; upregulation of (**F**) Catalase activity; (**G**) SOD activity and (**H**) GST. (**I**) *8l* treatment heightens expression of GST, TrxR, Nrf2, Keap1 and HSP60 in treated parasites. All the experiments were performed in triplicate and repeated for at least five times. ^*^p < 0.05 (One Way ANOVA) was considered statistically significant as compared to control.

We found a marked disruption in the redox homeostasis as pro-oxidants were found to be upregulated while cellular antioxidants were down regulated (Fig. 3). Significant upregulation in the total ROS and lipid peroxidation (as evident from the elevated level of MDA) alongside increased superoxide anion and hydrogen peroxide were observed after *8l* treatment (Fig 3A–D). In contrast, the level of GSH was down regulated (Fig. 3E). Key antioxidant enzymes of *S. cervi* viz. CAT, SOD and GST were elevated in *8l* treated parasites (Fig. 3F–H). On other side, upregulation of the expression of heat shock protein 60 (HSP 60), Nrf-2, thioredoxin reductase (TrxR) in *S. cervi* were noted (Fig. 3I). These changes collectively correlate with the increased abundance of the free radicals generated from oxidative damage to cellular biomolecules^24^. Therefore, *8l* potentially polarizes the filarial parasite towards a more pro-oxidative environment that diminishes their ability to survive by dampening the cellular anti-oxidants.

**Figure 4:**
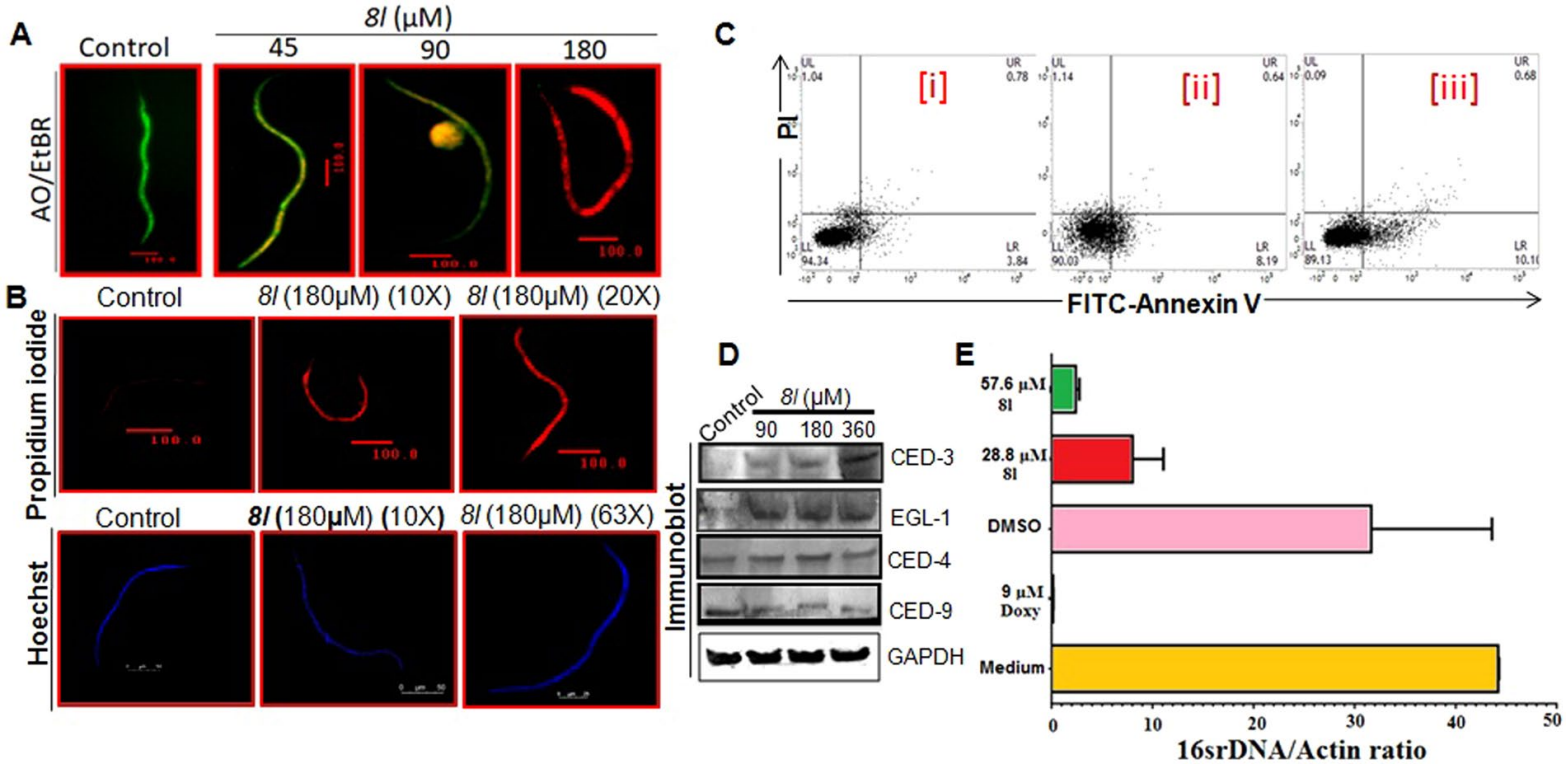
*8l* enhances apoptotic attributes in the developmental stages of *S. cervi* and exert anti-wolbachial activity. Microfilaricidal and ovicidal activity of *8l*. (**A**) Acridine orange (AO)/ethidium bromide (EtBr) double staining depicting occurrence of apoptosis in *S. cevi* microfilariae (Mf). (**B**) PI and Hoechst staining showing apoptotic death in Mf. (**C**) *8l* treatment induces expression of apoptosis associated proteins in Mf. (**D**) FITC-Annexin V/PI based flow cytometry showing quantitative proportions of different oocyte population viz. UL (necrosis), UR (late apoptosis), LL (viable cells), and LR (early apoptosis) treated with or without *8l*. [i] Represents control, [ii] and [iii] represent oocytes treated with *8l* with the doses of 18 µM and 36 µM, respectively. (**E**) *8l* has efficacy against *Wolbachia* endosymbionts. C6/36 cells infected with *Wolbachia* were incubated with 28.8 and 57.6 µM of *8l*, 9 µM of doxycycline, DMSO or untreated for 9 days. Cells were harvested and genomic DNA was extracted. *Wolbachia* depletion was monitored by qPCR using primers and hybridization probes specific for 16S rDNA (*Wolbachia*) and actin (insect cells). Endobacteria *16S rDNA* copies/µl were normalized to insect actin copies/µl. The bars show medians and the whiskers show interquartile ranges. Results are representative of three independent experiments repeated at least five times.

Besides adult parasites, we have also explored the earlier developmental stages of *S. cervi* viz. oocytes and Mf for deciphering the mode of action of *8l*. Our data on acridine orange (AO)/ethidium bromide (EtBr) and propidium iodide (PI) staining demonstrated induction of apoptosis alongside an increased generation of reactive oxygen species (ROS) with increasing doses of *8l* in treated Mf and oocytes (Fig. 4A; Supplementary Fig. 2B,C). Abundance of apoptotic cells in the oocytes and Mf were evidenced through differential AO/EtBr staining that showed a transition of green fluorescence towards red (Fig. 4 and Supplementary Fig. 2C), indicating damages to the membrane which is indicative of the induction of apoptosis^25^. Differential fluorescence staining using AO/EtBr indicated onset of apoptosis while increase PI staining intensity in the treated oocytes (Fig. 4B) resemble fragmented nuclear morphology (a cellular marker of apoptosis)^26^. FITC-annexin-V/PI based flow cytometric analysis confirmed induction of apoptosis with a gradual decrease in the live cell population (LL) and increase in early apoptotic cell population (LR) (3.84% for control, 8.19% for 18 µM and 10.10% for 36 µM) with the increase of treatment dose of *8l* (Fig. 4C). Such increment in the early apoptotic cell population with the abundance of *8l* in the incubation set up revealed that *8l* is a typical inducer of apoptosis and corroborates our previous findings^27,28^. However, the entire experimental group showed negligible percentage (~1%) of necrotic oocytes. Furthermore, upregulation of pro-apoptotic (EGL-1, CED-4 and CED-3) proteins and downregulation of anti-apoptotic protein CED-9 were evident in the treated Mf (Fig. 4D). Taken together, our experimental data suggested that oxidative stress mediated induction of apoptosis is central behind the antifilarial action of *8l* against all the developmental stages of *S. cervi* (Figs 2–4).

### 8l possesses potential anti-wolbachial activity

*8l* exposure resulted in a weak effect on the growth of *Wolbachia* in infected C6/36 insect cell line, but the endobacteria were depleted from the cells (Fig. 4E). *8l* resulted depletion of *Wolbachia* (16S rDNA/actin) in a dose dependent manner by more than a log (~90% reduction) compared to untreated infected C6/36 cells (Fig. 4E). No notable toxic effect on the insect cells was observed, as demonstrated by the expression of insect actin gene measured by quantitative real-time PCR and no change in the ability of the cells to grow was found (Fig. 4E).

### 8l is a benign molecule

*In vitro* toxicity study on RAW 264.7 cells revealed a high LC_50_ value (956.8 µM) that indicated towards non-toxic nature of *8l* (Supplementary Fig. 3). However, *in vivo* biotransformation can convert non-toxic moiety to a toxic one. Therefore we investigated the *in vivo* toxicity of *8l* in rat and found no toxic alterations in any of the toxicological parameters we studied (Supplementary Fig. 3). Histological observation of rat liver and spleen tissues demonstrated no notable changes in the tissue architecture (Supplementary Fig. 3). Haematological parameters viz. TC, DC, Hb etc. were close to the normal, and liver function parameters like SGOT, SGPT, ALP and bilirubin were at the level found in control animals (Supplementary Fig. 3).

## Discussion

This study is a maiden report on a novel class of antifilarial compound of which synthesis has been inspired from a very bioactive natural molecule belonging to quinolone based sulfa drugs. Sulfa drugs exist as the cyclic sulfonamides in nature, are heterocyclic compounds with diversified medicinal properties like antifungal, antibacterial, antiviral, antimicrobial, antimalarial, antineoplastic, antidiuretic, antiarrhythmic, insecticidal and sedative properties^9,19^. The wide spectrum of bioactivity of these compounds provided us the clue that these compounds can act over both unicellular as well as multicellular targets, prompted us to develop a hybrid molecule, i.e. coumarin and quinolone fused benzosultam, which may serve as a potential antifilarial drug without having any toxicity on non-targeted cells. Based on this postulation, *8l* was eventually screened from a series of twelve synthetic compounds with all desired attributes to be a potential antifilarial molecule.

In antifilarial drug development, the major setback has been the availability of a compound that acts against the adult parasite as most of the adulticidal drugs (e.g. suramin) are slow acting because they act on Wolbachial endosymbionts of the worm, or are too risky for use in MDA and risky even in a hospital setting^29^. Herein, we worked on the filarial worm *S. cervi* and *Wolbachia* separately. *S. cervi* is a non-*Wolbachia* containing worm and it is a WHO recommended model parasite due to its similarities with *W. bancrofti* in many aspects like antigenic patterns, nocturnal periodicity, metabolism etc.^23,30^. *8l* has lethal action against adults, Mf and oocytes with good LD_50_. Ideally, new antifilarial drugs should be macrofilaricidal; therefore the molecular mode of action of *8l* on the adult stage filarids was emphasized with the results that *8l* indeed is a potent macrofilaricidal agent.

Whilst studying the molecular mechanism of action our experimental data primarily demonstrated that *8l* results in death of the filarial nematode *S. cervi* through induction of programmed cell death via the Egl-1/Ced-4/Ced-3 cascade. The primary signal behind inducing apoptosis was found to be ROS generated from the peroxidation of membrane lipids. Alongside ROS, *8l* physically interacts with the minor groove of parasitic DNA, induces DNA fragmentation and suppresses DNA repair machinery which signals activation of p53 and finally CED-3, the nematode effector caspase. p53 has been reported to play a critical role in ROS mediated apoptosis especially wherein DNA damage occurs^31^. Recently we had reported on the activation of the CED pathway in the context of *S. cervi* apoptosis^23,26^, but here we have shown that p53 appears to play a role in transmitting the apoptotic signal for activating the CED cascade. Downstream activation of PARP through proteolytic cleavage by caspase-3 designated activation of intrinsic pathway that most likely cross-talks with the CED pathway to mediate cell death in *S. cervi*.

Efficacy of *8l* as microfilaricide prompted us to investigate the possible activity of *8l* as an anti-wolbachial agent. The concentrations of *8l* tested were not lethal to the C6/36 cells infected with *Wolbachia* but the endobacteria were depleted from the cells in a dose dependent manner. This specific action against *Wolbachia* revealed that *8l* could be very effective in treating the human filarial infections *W. bancrofti, O. volvulus, Brugia spp*. and some *Mansonella spp*., due to their dependence on the endosymbionts for survival. Having multiple targets in the nematodes could reduce the chances of developing resistance to this compound. Moreover, our preclinical toxicity studies comprising haematological, serological and biochemical investigation revealed no toxic alteration in the *8l* treated rat groups. Therefore, *8l* is not only an efficacious antifilarial agent and but also is safe for possible development as a drug for treating lymphatic filariasis.

## Methods

### Reagents

All culture related materials were obtained from Sigma Aldrich, St. Louis MO, USA. HPLC grade methanol, acetic acid and other chemicals and solvents of highest purity grade were purchased from Merck, India. Primary antibodies and Horseradish Peroxidase labeled secondary antibody were purchased from Santa Cruz Biotechnology (Santa Cruz, CA).

### Synthesis and characterization of the sulphonamide

The benzosultam, 4,7-dimethyl-3,4,7,8-tetrahydro-3λ^6^-[1,2]thiazino[4,3-*f*]quinoline-3,3,8-trione (*8l*) was synthesized from its precursor quinolone (6-amino-5-bromo quinolone derivative) with the reaction of 2-chloroethylsulfonyl chloride at 0 ^o^C followed by Heck cyclization as described in our earlier report^20^. After synthesis of the compound *8l*, the product was purified by column chromatography using silica gel (60–120 mesh size) as column matrix and eluted with 30% ethyl acetate–petroleum ether to afford the compound *8l* as a white solid. Furthermore, the compound was crystallized from chloroform and the pure single crystal of *8l* was obtained with a melting point of 246–248 °C. The purified compound (*8l*) was characterized by ^1^H & ^13^C NMR, DEPT-135 NMR, mass spectroscopy, CHN-analysis and IR spectroscopic analysis (detail has been demonstrated in Mondal *et al*.^20^). No impurity was found in the purified *8l* in any of the analysis^20^. The molecular weight of *8l* was found to be 276.31 through mass spectroscopic analysis^20^. Summary of the procedure of the synthesis of *8l* is given in Supplementary Fig. 1A.

### Preparation of sample

The compound 4,7-dimethyl-3,4,7,8-tetrahydro-3λ^6^-[1,2]thiazino[4,3-*f*]quinoline-3,3,8-trione (*8l****)*** was dissolved in DMSO (Merck, Germany) to prepare a stock and thereafter diluted in RPMI dissolved in Milli-Q water to yield the desired concentrations (4.5–360 µM) to be used in the study. Before applying to the biological system, the solution of *8l* was filtered through a 0.22 µm membrane filter (Millipore, USA).

### Parasite culture and treatments

*Setaria cervi* Rudolphi (Onchocercidae), a model filarial parasite was used in the study. Adult filarids were obtained from the peritoneal cavity of freshly slaughtered cattle at local abattoirs and collected in Kreb’s Ringer bicarbonate buffer (Sigma). Microfilariae (Mf) and oocytes (early and late embryonic stages) were obtained from the uterus of dissected gravid female parasites as described previously^27^. Oocytes (1.0 × 10^6^), Mf (1.0 × 10^4^) and adults (1 male and 1 female) of *S. cervi* were separately incubated with different doses of *8l* (4.5–360 µM) in sterile tissue culture flasks. Each incubation experiments was set in triplicate and replicated for five times.

### MTT reduction assay

Viability of *8l* treated parasites was determined by MTT (3-(4,5-dimethyl-thiazol-2-yl)-2,5-diphenyltetrazolium bromide) reduction assay and LC_50_ values were calculated as described earlier^27^. In brief, control and treated parasites from the incubation media were harvested through centrifugation at 5000 × g for 5 min. Parasites were washed with PBS (50 mM, pH 7.4), suspended in 500 μl of MTT (0.5 mg/ml in PBS) and incubated for 2 hr at 37 ^ο^C at dark. The dark formazan crystals were solubilized by adding 100 μl of DMSO and the color intensity was measured at 595 nm using a microplate reader (Bio-Rad, USA). Each experiment was conducted in triplicate and repeated for at least five times.

### Histology and Hematoxylin-Eosin staining

Following incubation with *8l*, both the treated and control parasites were prepared for ultra-microtomy and stained with Hematoxylin-Eosin dye following the procedure of Joardar *et al*.^32^. In brief, parasites were fixed in 4% paraformaldehyde (prepared in chilled PBS) for 12 hr, washed in PBS and subjected to dehydration ethanol gradation (30–100%). Dehydrated parasite tissues were incubated in absolute xylene for 2 min followed by incubation in xylene: paraffin (1:1) and finally embedded in paraffin (Merck, India). Paraffinized tissues were subjected to ultra-microtomy to obtain tissue sections with thickness of 5 µm. Tissue sections in the paraffin ribbon were stretched, deparaffinized and stained with Hamatoxylin-Eosin dye (Merck, India). Stained tissue sections were mounted on a clean glass slide using DPX (Merck, India) and examined with a bright-field optical microscope (Dewinter, Italy).

### Microscopic determination of apoptosis

Propidium iodide (PI) staining was performed following the procedure depicted in Saini *et al*.^33^. Oocytes were incubated with different concentrations (9 to 36 μM) of *8l* for 6 hr. Test samples were washed with Hank’s balanced salt solution (HBSS), permeabilized with acetone: methanol (1:1 v/v), washed with HBSS and incubated with propidium iodide (70 μM) for 15–30 min in the dark. Alteration in the control and treated samples were examined with a fluorescence microscope (Dewinter, Italy).

Control and *8l* treated oocytes and Mf were subjected to acridine orange (AO)-ethidium bromide (EtBr) double staining following Dey *et al*.^25^. In brief, AO and EtBr (150 μM) were prepared in 0.1 M PBS in separate vials and mixed together 1:1 v/v before adding to the suspension of oocytes or Mf. Staining was conducted for 10 min at room temperature in the dark, mounted over a poly-L-lysine coated glass slide and examined with a fluorescence microscope (Dewinter, Italy).

Changes in nuclear morphology were studied by Hoechst staining as described in Mukherjee *et al*.^27^.Histological sections of parasite tissues were mounted on glass slides, de-paraffinized using xylene and rehydrated in phosphate buffer saline (PBS). Sections were stained with 1 µM bisbenzimide solution (Hoechst staining solution; Sigma-Aldrich), incubated at room temperature for 5 min and then examined under a fluorescence microscope (Carl Zeiss) using an excitation wave length of 495 nm.

### Caspase Assay

Caspase activity in the parasite homogenate was determined using the microplate based CaspACE™ Assay System (Promega, USA) following manufacturer’s guidelines. *P*-nitroaniline labeled DEVD peptide was used as substrate while Z-VAD-FMK was used as inhibitor.

Immunofluorescence of CED-3 (nematode caspase-3 homolog) was performed following the method described in Joardar *et al*.^32^. In brief, deparaffinized histological sections of control and *8l* treated *S. cervi* were blocked with 1% BSA in PBST (PBS containing 0.1% Tween 20) for 1 hr. Blocked tissue sections were incubated with nematode specific anti-CED3 antibody (1:200 dilution in 1% BSA in PBST) for 6 hr in a humidified chamber followed by incubation with FITC-labeled IgG (1:500 in 1% BSA in PBST) for 2 hr in the dark. Slides were washed twice with PBST and examined under a fluorescence microscope (Dewinter, Italy).

### Immunoblotting

Immunoblotting was performed following the method depicted in our previous report by Mukherjee *et al*.^27^. Briefly, adult parasites were homogenized in CellLytic^TM^ lysis buffer (Sigma) for 10 mins at room temperature, subjected to ultrasonication (60 mHz, 3 pulses for 10 sec each) and centrifuged for 20 mins at 10000 × g. The clear supernatant obtained after centrifugation was treated with Proteoblock^TM^ protease inhibitor cocktail (Fermentas, USA) and used for immunoblotting.

Total protein content was determined by the Lowry method^34^. 70 µg of protein from each cell free supernatant was resolved by 12.5% SDS-PAGE, electro-transferred onto PVDF membrane and probed with specific primary antibodies followed by alkaline phosphatase or HRP labeled IgG for 6 hr. Protein bands were visualized using BCIP-NBT and photographed with a Chemidoc system (Bio-Rad, USA).

### DNA fragmentation assay

Fragmentation in genomic DNA of control and treated adult parasites was studied following Mukherjee *et al*.^27^. For isolation of genomic DNA, control and treated parasites were homogenized in 500 μl lysis buffer (20 mM Tris-HCl, pH 8.0; 50 mM EDTA; 0.5% SDS; 100 mM NaCl; 0.1% β-mercaptoethanol and proteinase-K (0.1 mg/ml)) and incubated in water bath for 2 hr at 37 ^ο^C. Genomic DNA was extracted using a mixture of phenol:choloroform: iso-amyl alcohol (25:24:1) and DNA was precipitated by adding cold ethanol (95%). The DNA pellets were air dried and solubilized in 20 μl of Tris-EDTA buffer (10 mM tris-HCl and 1 mM EDTA; pH 8.0). Each purified DNA sample (7 μg) was resolved by 1.8% agarose gel electrophoresis (at 50 V for first 15 min then 100 V for 90–100 min), stained with ethidium bromide (EtBr) and visualized in a Gel-Doc (Bio-Rad, USA).

### Flow cytometry

To present quantitative data on the pro-apoptotic effect of 8l in *S. cervi*, oocytes were treated with variable doses of *8l* (1.8 and 3.6 µM) for 24 hr at 37 °C and were harvested by centrifuging at 2275 × g for 5 min. Oocytes were stained with annexinV-FITC and propidium iodide using the Apoptosis Detection Kit (Sigma) following manufacturer’s guidelines. Flow cytometry was performed using FACSverse cytometer equipped with FACSDiva Version 6.1.2 software (BD biosciences).

### Circular Dichroism (CD) spectroscopic analysis

To determine the physical interaction between *8l* and genomic DNA of the parasite, CD spectroscopy was performed following the procedure described earlier^25^. In brief, isolated genomic DNA of *S. cervi* was prepared in an aqueous solution to yield a concentration of 1 mg/ml wherein three different doses of *8l* (36, 90 and 180 µM) were added in separate reactions set alongside a control. The instrument was operated at a scanning speed of 50 nm/min, bandwidth of 10 nm and sensitivity of 100 millidegrees. The molar ellipticity values are expressed in terms of the mean residue molar ellipticity.

### Determination of oxidative stress

Induction of oxidative stress in parasites was determined by H2DCFDA assay following Wojtala *et al*.^35^. In brief, control and *8l* treated parasites were with 0.4 mM H2DCFDA for 15 min in the dark. Parasites were washed in PBS, lysed using cell lysis buffer (Cell Lytic^TM^) and the fluorescence intensity of the supernatant was measured using a spectrofluorimeter (LS55, Perkin Elmer) with an excitation wavelength of 504 nm and emission wavelength of 529 nm. Each experiment was performed in triplicate and replicated for five times.

Malondialdehyde (MDA) level in the control or *8l* treated parasites was estimated by the thiobarbituric acid reaction method following Mukherjee *et al*.^9^. In brief, samples were de-proteinized by treating with trichloroacetic acid and dissolved in HCl. Thiobarbituric acid was added immediately, boiled for 10 mins in a water bath and brought to room temperature. Each sample was centrifuged at 10,000 × g for 15 mins, supernatant was collected and color intensity was recorded at 532 nm using a dual-beam spectrophotometer (Shimadzu, Japan). MDA concentration in the test sample was calculated by the following equation:

$${\rm{Malondialdehyde}}\,{\rm{concentration}}\,({\rm{M}})={\rm{Absorbance}}\,{\rm{at}}\,{\rm{532}}\,{\rm{nm}}/{\rm{\varepsilon }}\,{\rm{and}}\,{\rm{expressed}}\,{\rm{as}}\,\mu \mathrm{mol}/{\rm{mg}}\,{\rm{protein}}{\rm{.}}$$

(ε = Extinction coefficient 1.56 × 10^5^).

Superoxide anion (O_2_^−^) level was determined by nitro blue tetrazolium (NBT) assay following Saini *et al*.^26^. In brief, control and treated parasites were incubated with 2% NBT (prepared in PBS) for 1 hr at room temperature, washed with PBS and fixed in methanol. The resultant dark formazan crystals were dissolved in a mixture of 240 μl of 2 M KOH and 480 μl of DMSO. The absorbance was measured at 620 nm using a microplate reader (Bio-Rad, USA).

Level of H_2_O_2_ in the control and treated *S. cervi* homogenates was estimated according to Mukherjee *et al*.^23^ with modifications. 50 μl of parasite homogenate was mixed with 450 μl of distilled water and added to 1 ml dichromate/acetic acid solution (5% potassium dichromate in glacial acetic acid 1:3 v/v). The mixture was incubated in a water bath for 10 min, brought to room temperature and optical density (O.D.) was measured at 570 nm.

### Assay of reduced glutathione and activities of the antioxidant enzymes

Reduced glutathione (GSH), Superoxide dismutase (SOD), Catalase, and glutathione S-transferase (GST) activities in the lysates of control and *8l* treated filarids were determined according to our previous reports^26^. Control and treated parasites were homogenized in cell lysis buffer (Cell Lytic ^TM^) supplemented with protease inhibitor cocktail (Fermentas, USA). The homogenate was centrifuged at 15,000 × g for 30 min at 4 ^ο^C and clear supernatant was used for the experiments.

Level of GSH was estimated following Mukherjee *et al*.^27^. 150 μl of tissue homogenate was mixed with 5% perchloric acid and centrifuged at 1000 × g for 10 min at 4 ^ο^C. 100 μl of clear supernatant was added to a mixture of 1.88 ml of 0.1 M potassium phosphate buffer (pH 8.0) and 20 μl of DTNB (5,5-dithio-bis-(2-nitrobenzoic acid). After an incubation of 3 min at 25 ^ο^C, the absorbance was recorded at 412 nm using a spectrophotometer (Shimadzu 1601 UV-Vis spectrophotometer). Level of GSH was expressed in nanomole/mg protein.

GST activity was determined in the cell free supernatant following method of Mukherjee *et al*.^27^. 100 μl of cell free supernatant was added to 1 ml of assay cocktail comprising 980 μl of PBS (pH 6.5, 100 mM CDNB (1-chloro-2,4-dinitrobenzene) and 100 mM GSH. The assay mixture was incubated at 30 ^ο^C for 5 min and absorbance was measured at 340 nm. Enzyme activity was expressed at U/mg protein.

Catalase activity was assayed following the method described in Mukherjee *et al*.^27^; Mukherjee *et al*.^31^. 40 μl of cell free supernatant from the parasite homogenate was added to the assay mixture containing 3 ml of H_2_O_2_-phosphate buffer (2 mM H_2_O_2_ prepared in 0.067 M PBS (pH 7.0)) and mixed vigorously. The absorbance was read at 240 nm using a UV-VIS spectrophotometer (Shimadzu, Japan). Specific activity of the enzyme was expressed in U/mg protein.

SOD activity was also determined in the supernatant of parasite homogenate using the SOD assay kit (Cayman Chemical, USA) following the manufacturer’s guidelines. The enzyme activity was assayed by measuring the formation of formazan dye by the action of SOD and the color intensity was recorded spectrophotometrically at 495 nm. The enzyme activity was expressed as U/mg protein.

### Determination of anti-wolbachial activity of 8l

*Wolbachia* infected C6/36 insect cells were treated for 9 days with different concentrations of the compound (28.8 μM and 57.6 μM), 10% DMSO vehicle, medium or Doxycycline (9 μM) following Schiefer *et al*.^28^. DNA was extracted and the *Wolbachia* content was measured by qPCR of the *16S rRNA* gene normalized to the insect *actin* gene.

### Assessment of toxicity of 8l in rat model

Toxicity of *8l* was tested in macrophage cell line (RAW 264.7) and animal model (rat) following our earlier reports^25,31^.

For *in vitro* toxicity evaluation, 1 × 10^6^ mouse macrophages (RAW 264.7, ATCC, USA) were cultured in six well plates (Nest Biotechnology) with incomplete media (DMEM) containing 100 U/ml penicillin and 100 μg/ml streptomycin at 37 ^ο^C in 5% CO_2_ atmosphere following Mukherjee *et al*.^36^. Cells were treated with different concentrations (450 and 900 μM) of *8l* for 24 hr. After incubation, cells were isolated by scrapping and centrifuged to remove the spent medium. Cell pellet was washed twice with PBS and cell viability was determined by MTT assay as depicted in earlier section.

For *in vivo* toxicity analysis, *8l* was administered orally (10 and 25 mg/kg body weight) to Wister rat (130 ± 5 gm) for a period of seven days. After completion of treatment, rats were euthanized, the livers and spleens were perfused with PBS and processed for histological preparation. Liver and spleen tissues were fixed in Bouins fixative for at least 24 hrs, dehydrated with graded alcohol, embedded in paraffin and subjected to ultramicrotomy.

The protocols of the experiments conducted with the laboratory animals were approved by Institutional Animal Ethical Committee (Ref. No. IAEC/VB/2016/11 dated 20/08/2016) and performed according to the guidelines of Committee for the Purpose of Control and Supervision of Experiments of Animals (CPCSEA); Govt. of India (Registration number: 1819/GO/Ere/S/15/CPCSEA) and OECD guidelines (OECD, 2017)^37^. The animals had free access to food and water, and were maintained under controlled temperature (27 ± 2 °C) and 12 h light and 12 h dark cycle. The initial body weights of the animals were recorded. At the end of the study, rats were deprived of food overnight, anesthetized and blood was collected by cardiac puncture (1 ml into EDTA for hematology, and 2 ml for clinical biochemistry). Blood was centrifuged at 1500 × g for 15 min, and the plasma was immediately collected. All samples were processed within 4–6 hours. Detail on the experimental procedure has been provided in Supplementary Material.

### Statistical analyses

All the experiments were performed in triplicate and repeated at least five times. All the experimental data are represented as mean ± SD. Significant differences (*p* value < 0.05) between the means of control and *8l*-treated parasites were analyzed by One-way ANOVA with Tukey’s post-hoc test using GraphPad Prism 5.0.

## Conclusion

Sulfonamides have well known antibacterial efficacy, principally as blockers of folate metabolism. This study is a first report on the potency and safety of a synthetic sulfonamide as an antifilarial agent effective against all the developmental stages of the parasite and against the endosymbiotic bacteria. Therefore, its serum availability, pharmacokinetics and pharmacodynamics should be studied to provide data necessary for proceeding towards clinical trials.

## Electronic supplementary material

Supplementary Information

## References

[1] World Health Organization (WHO). Lymphatic filariasis. www.who.int/mediacentre/factsheets/fs102/en/ Accessed 6 December 2017.

[2] Mukherjee S, Mukherjee N, Gayen P, Roy P, Sinha Babu SP (2016). Metabolic inhibitors as antiparasitic drugs: pharmacological, biochemical and molecular perspectives. Curr. Drug Metab..

[3] Specht S (2013). Chemotherapy of filariasis- established strategies and new developments. GMS Infect. Dis..

[4] Taylor MJ, Hoerauf A, Bockarie M (2010). Lymphatic filariasis and onchocerciasis. The Lancet.

[5] Turner JD (2009). *Wolbachia* lipoprotein stimulates innate and adaptive immunity through Toll-like Receptors 2 and 6 to induce disease manifestations of filariasis. J. Biol. Chem..

[6] Taylor MJ (2005). Macrofilaricidal activity after doxycycline treatment of *Wuchereria bancrofti*: a double blind, randomised placebo-controlled trial. The Lancet.

[7] Debrah AY (2006). Doxycycline reduces plasma VEGF-C/sVEGFR-3 and improves pathology in lymphatic filariasis. PLoS Pathog..

[8] Taylor MJ, Hoerauf A, Townson S, Slatko BE, Ward SA (2014). Anti-Wolbachia drug discovery and development: safe macrofilaricides for onchocerciasis and lymphatic filariasis. Parasitol..

[9] Li B (2015). New synthesis method for sultone derivatives: synthesis, crystal structure and biological evaluation of S-CA. Molecules.

[10] De Castro S (2011). From β-amino-g-sultone to unusual bicyclic pyridine and pyrazine heterocyclic systems: Synthesis and cytostatic and antiviral activities. Chem Med Chem..

[11] Babaoglu K, Qi J, Lee RE, White SW (2004). Crystal structure of 7,8-dihydropteroate synthase from Bacillus anthracis: mechanism and novel inhibitor design. Structure.

[12] Mastrolorenzo A, Scozzafava A, Supuran CT (2000). Antifungal activity of silver and zinc complexes of sulfadrug derivatives incorporating arylsulfonylureido moieties. Eur. J. Pharm. Sci..

[13] Bar G, Parsons AF, Thomas CB (2001). Manganese(III) acetate mediated radical reactions leading to araliopsine and related quinoline alkaloids. Tetrahedron.

[14] Lee YR, Kim BS, Kweon HI (2000). Efficient synthesis of dihydrofuroquinolinones and furoquinolinones by silver(I)/celite promoted oxidative cycloaddition. Tetrahedron.

[15] Pirrung MC, Blume F (1999). Rhodium-mediated dipolar cycloaddition of diazoquinolinediones. J. Org. Chem..

[16] Lebegue N (2005). Novel benzopyridothiadiazepines as potential active antitumor agents. J. Med. Chem..

[17] Wells GJ, Tao M, Josef KA, Bihovsky R (2001). 1,2-benzothiazine 1,1-dioxide P2−P3 peptide mimetic aldehyde calpain I inhibitors. J. Med. Chem..

[18] Supuran CT (2008). Carbonic anhydrases: novel therapeutic applications for inhibitors and activators. Nat. Rev. Drug Discov..

[19] Majumdar KC, Mondal S (2011). Recent developments in the synthesis of fused sultams. Chem. Rev..

[20] Mondal S, Debnath S, Pal S, Das A (2015). Synthesis of uracil-, coumarin-and quinolone-fused benzosultams and benzosultones. Synthesis.

[21] Mondal, S., *et al*. Studying the biological activities and molecular docking of some novel benzosultams and benzosultones. *Curr.Bioact. Comp*.**12**, 10.2174/1573407212666161028160745 (2017).

[22] Schellenberg MJ, Williams RS (2011). DNA end processing by polynucleotide kinase/phosphatase. Proc. Natl. Acad. Sci. USA.

[23] Mukherjee N (2016). Oxidative stress plays major role in mediating apoptosis in filarial nematode *Setaria cervi* in the presence of trans-stilbene derivatives. Free Radic. Biol. Med..

[24] Sampayo JN, Olsen A, Lithgow GJ (2003). Oxidative stress in *Caenorhabditis elegans*: protective effects of superoxide dismutase/catalase mimetics. Aging Cell.

[25] Dey B (2016). Green silver nanoparticles for drug transport, bioactivities and a bacterium (*Bacillus subtilis*)-mediated comparative nano-patterning feature. RSC Adv..

[26] Saini P (2014). Antifilarial effect of ursolic acid from *Nyctanthes arbortristis*: molecular and biochemical evidences. Parasitol. Int..

[27] Mukherjee N, Mukherjee S, Saini P, Roy P, Sinha Babu SP (2014). Antifilarial effects of polyphenol rich ethanolic extract from the leaves of *Azadirachta indica* through molecular and biochemical approaches describing reactive oxygen species (ROS) mediated apoptosis of *Setaria cervi*. Exp. Parasitol..

[28] Schiefer A (2013). The ClpP peptidase of *Wolbachia* endobacteria is a novel target for drug development against filarial infections. J. Antimicrob. Chemother..

[29] Pfarr KM, Hoerauf AM (2006). Antibiotics which target the *Wolbachia* endosymbionts of filarial parasites: a new strategy for control of filariasis and amelioration of pathology. Mini Rev. Med. Chem..

[30] Kaushal NA, Kaushal DC, Ghatak S (1987). Identification of antigenic proteins of *Setaria cervi* by immunoblotting technique. Immunol. Invest..

[31] Mukherjee S, Mukherjee N, Saini P, Roy P, Sinha Babu SP (2015). Ginger extract ameliorates phosphamidon induced hepatotoxicity. Indian J. Exp. Biol..

[32] Joardar N, Mukherjee S, Sinha Babu SP (2018). Thioredoxin reductase from the bovine filarial parasite *Setaria cervi*: Studies on its localization and optimization of the extraction. Int. J. Biol. Macromol..

[33] vickSaini P (2012). Effect of ferulic acid from *Hibiscus mutabilis* on filarial parasite *Setaria cervi*: molecular and biochemical approaches. Parasitol. Int..

[34] Lowry O, Rosebrough NJ, Farr AL, Randall RJ (1951). Protein measurement with the folin phenol reagent. J. Biol. Chem..

[35] Wojtala A (2014). Methods to monitor ROS production by fluorescence microscopy and fluorometry. Methods Enzymol..

[36] Mukherjee S, Mukherjee S, Maiti TK, Bhattacharya S, Sinha Babu SP (2017). A novel ligand of Toll-like receptor 4 from the sheath of *Wuchereria bancrofti* microfilaria induces proinflammatory response in macrophages. J. Infect. Dis..

[37] Organisation for Economic Cooperation and Development (OECD) Environment, Health and Safety Publications Series on Testing & Assessment No. 261 Paris (2017).

