# Supplementary material for: Quinolone-fused cyclic sulfonamide as a novel benign antifilarial agent
Source: Sci Rep. 2018 Aug 13;8:12073. doi: 10.1038/s41598-018-30610-7 (PMC6089915; doi:10.1038/s41598-018-30610-7)
Supplement: Supplementary file 1 — Supplementary Information [file 41598_2018_30610_MOESM1_ESM.docx]

**Supplementary Information**

**Quinolone-fused cyclic sulfonamide as a novel benign antifilarial agent**

Suprabhat Mukherjee^1^, Nikhilesh Joardar^1^, Shovan Mondal^2^, Andrea Schiefer^3^, Achim Hoerauf^3^, Kenneth Pfarr^3,4*^ and Santi P. Sinha Babu^1*^

^1^Department of Zoology, Visva-Bharati University, Santiniketan 731 235, India

^2^Department of Chemistry, Syamsundar College, Shyamsundar-713 424, India

^3^Institute of Medical Microbiology, Immunology and Parasitology, University Hospital Bonn, ^4^German Center for Infection Research (DZIF), D-53127 Bonn, Germany

^*^Corresponding authors. E-mail: [kenneth.pfarr@ukbonn.de](mailto:kenneth.pfarr@ukbonn.de) (KP); [spsinhababu@gmail.com](mailto:spsinhababu@gmail.com) (SPS)

**
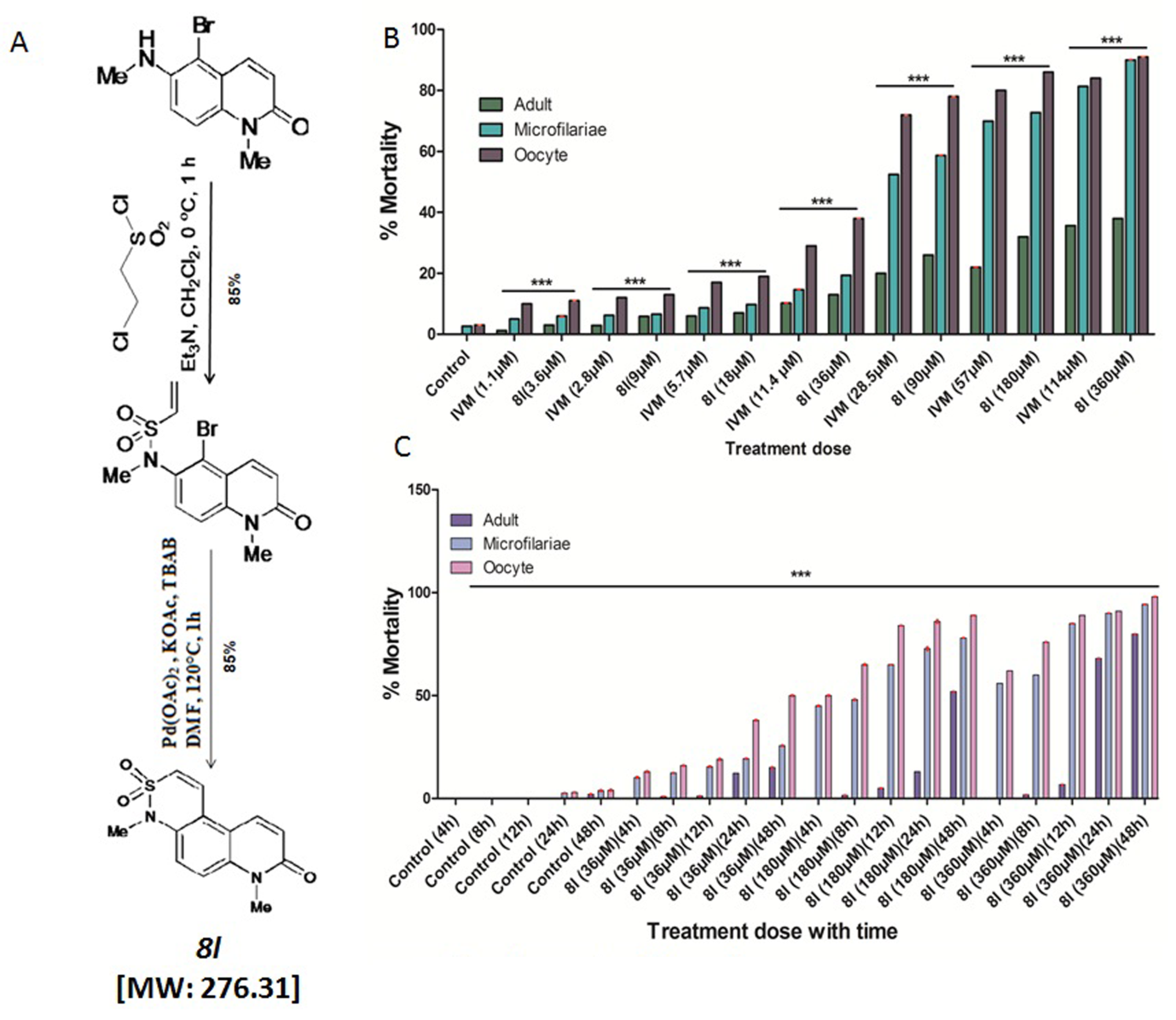
**

**Supplementary Figure 1**. Synthesis and antifilarial activity of *8l*. A. Scheme depicting the procedure of the synthesis of *8l* i.e. 4,7-dimethyl-3,4,7,8-tetrahydro-3λ^6^-[1,2]thiazino[4,3-*f*]quinoline-3,3,8-trione. The benzosultam,4,7-dimethyl-3,4,7,8-tetrahydro-3 λ^6^-[1,2]thiazino[4,3-*f*]quinoline-3,3,8-trione (**3**) was prepared from 6-amino-5-bromo quinolone derivative (**1)** with the reaction of 2-chloroethylsulfonyl chloride at 0^o^C followed by Heck cyclization. This synthesized compound was characterized by ^1^H &^13^C NMR, mass, CHN-analysis and IR spectroscopic analysis. The details of the synthetic procedure and the characterization of compound **3** have been described previously by Mondal *et al*.^20^. B & C. Dose and time dependent antifilarial activity of *8l* against oocytes, Mf and adult stage of the filarial parasite, *S. cervi*. Experiments were performed in triplicate and repeated for at least five times. All the data were presented as mean±SD and **p*<0.05 was considered as statistically significant.


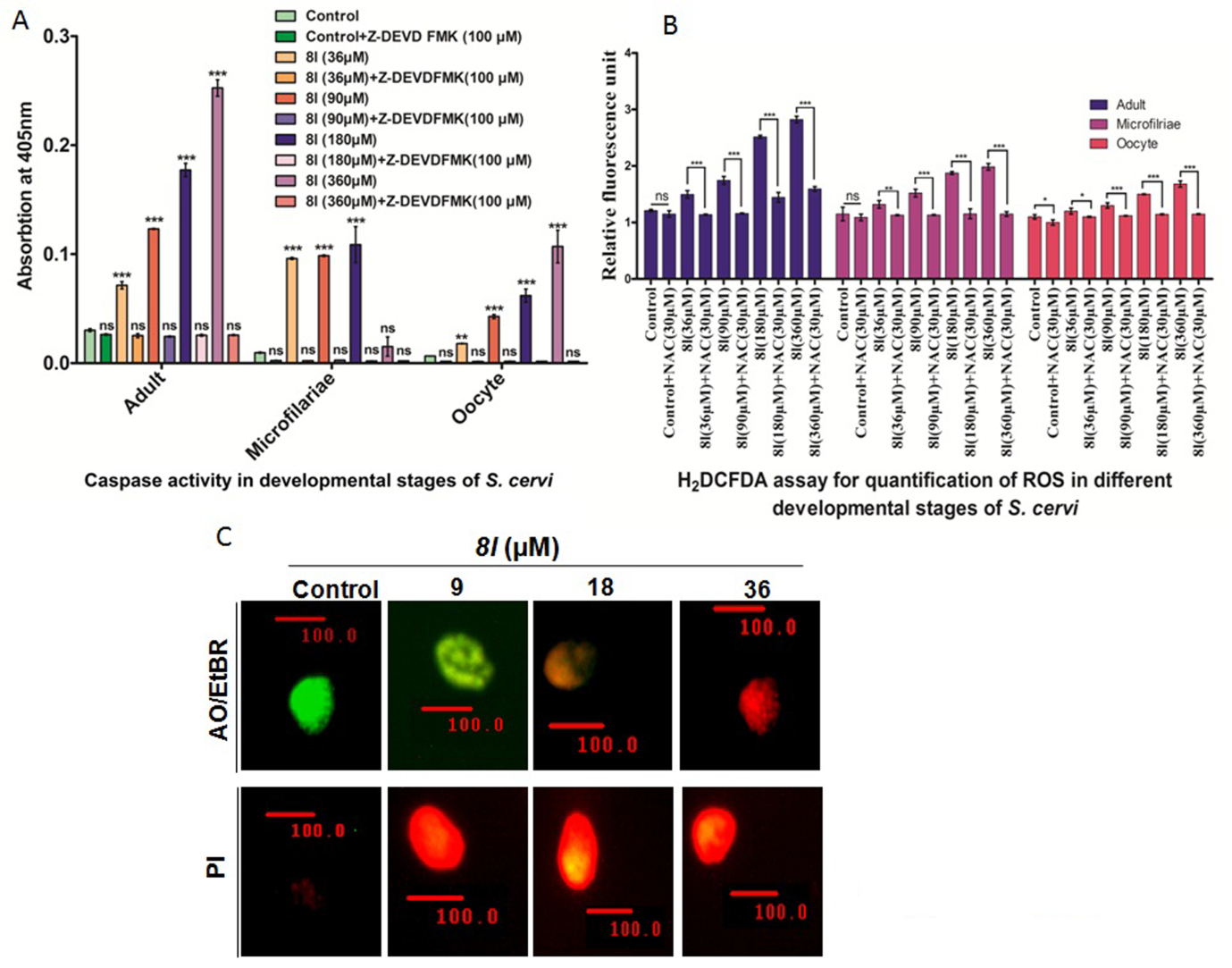


**Supplementary Figure 2**. Molecular mechanism of action of *8l*. A. *8l* induced caspase activation amongst the developmental stages of *S. cervi*. *P*-nitroaniline labeled DEVD peptide was used as the substrate while Z-DEVD-FMK was used as the inhibitor. B. Generation of ROS in the *8l* treated oocytes, Mf and adults. In the incubation vessel, adults, Mf and oocytes were incubated in 10 ml and 2 ml of culture media, respectively^23,27^. Each experiment was performed in triplicate and repeated at least five times. All the data were presented as mean±SD. C. Mechanism of ovicidal activity of *8l*. Acridine orange (AO)/ethydium bromide (EtBr) double staining (upper panel) and propidium iodide (PI) staining (lower panel) indicating induction of apoptosis in oocytes.In brief, oocytes were incubated with different concentrations (18 and 36 μM) of *8l* for 6 hours. Test samples were washed with Hank’s balanced salt solution (HBSS), permeabilized with acetone: methanol (1:1 v/v), washed with HBSS and incubated with propidium iodide (70 μM) for 15-30 min in the dark. Alteration in the control and treated samples were examined with a fluorescence microscope (Dewinter, Italy). In brief, AO and EtBr (150 μM) were prepared in 0.1 M PBS in separate vials and mixed together 1:1 v/v before adding to the suspension of oocytes or Mf. Staining was conducted for 10 min at room temperature in the dark, mounted over a poly-L-lysine coated glass slide and examined with a fluorescence microscope (Dewinter, Italy). Each micrograph is a representative of five independent experiments.


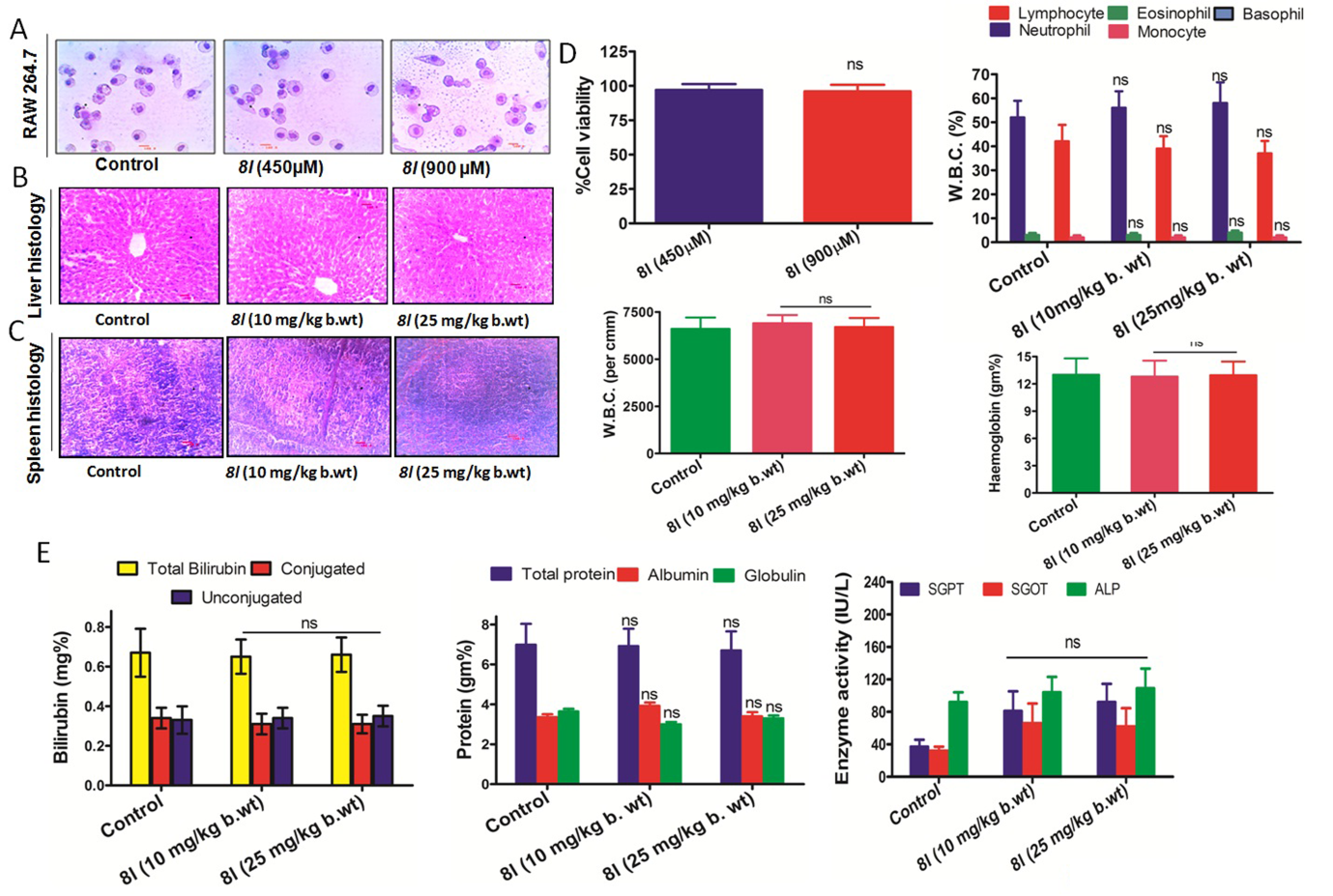


**Supplementary Figure 3**. Analyses of the toxicity of *8l in vitro* and *in vivo.* A. Bright field micrographs showing the effect of *8l* on the viability of RAW 264.7 macrophages *in vitro*. Histogram depicting the result of MTT assay for determining the viability of the macrophages after *8l* treatment. RAW 264.7 macrophages were treated with *8l* with indicated doses and analyzed for cell viability and cellular morphology. Cell viability was monitored by MTT assay and phase contrast microscopy as described in Dey *et al*.^25^. B. and C. Histological examination of liver and spleen tissues of control and *8l* treated rat. Histological observation of rat liver and spleen tissues demonstrated no notable change in the tissue architecture. Freshly perfused liver tissues were fixed in Bouin's fixative overnight, dehydrated in an ethanol series, and embedded in paraffin for histological procedures. Paraffinized sections (thickness 5 μm) were stained with Haematoxylin and Eosin (HE). Each slide was assessed for specific histological alterations under an inverted light microscope (Dewinter, Italy). Changes in D. haematological parameteters, E. serological and enzymological parameters. Haematological parameters viz. TC, DC, Hb etc. (D) were close to the normal, and liver function parameters like SGOT, SGPT, ALT and bilirubin all were also at the level found in the control animals (E). Biochemical analyses included measurement of serum total protein, albumin, globulin, total bilirubin both conjugated and un-conjugated, serum glutamate oxaloacetate transaminase (SGOT), serum glutamate pyruvate transaminase (SGPT) and alkaline phosphatase following Mukherjee *et al*.^33^. Haematological parameters viz. leukocyte contents, haemoglobin content, etc., are critical to physiological homeostasis^33^. Similarly, activity of serum transaminases like SGOT (Serum glutamate oxaloacetate transaminase), SGPT, ALP etc. are the crucial indicators of liver function^25, 33^. Herein, no sign of hepatic dysfunction was measured as all liver function markers were within the normal range. Moreover, other biochemical parameters were also found to be normal. For i*n vivo* toxicity analyses, *8l* was administered orally (10 and 25 mg/kg body weight) to Wister rats (130±5 gm) for a period of 7 days. After completion of treatment, rats were euthanized; the livers and spleens were perfused with PBS and processed for histological preparation. Liver and spleen tissues were fixed in Bouins fixative for at least 24 hrs, dehydrated with graded alcohol, embedded in paraffin and subjected to ultramicrotomy. All the experiments were performed in triplicate and repeated for at least five times. Each data represent mean±SD. **p*<0.05 considered as statistically significant.
